# Assessing the Determinants of Compliance with Contribution Payments to the National Health Insurance Scheme among Informal Workers in Indonesia †

**DOI:** 10.3390/ijerph20237130

**Published:** 2023-11-30

**Authors:** Trisnasari, Orapin Laosee, Cheerawit Rattanapan, Piyapong Janmaimool

**Affiliations:** 1 ASEAN Institute for Health Development, Mahidol University, 999 Salaya, Phuttamonthon, Nakhon Pathom 73710, Thailand; tars122021@gmail.com (T.);; 2 Social Security Administrator for Health (BPJS Kesehatan), Jakarta 10150, Indonesia

**Keywords:** informal workers, National Health Insurance, payment compliance, universal health coverage, social health protection

## Abstract

This study aimed to investigate the determinants of compliance with contribution payments to the National Health Insurance (NHI) scheme among informal workers in Bogor Regency, West Java Province, Indonesia. Surveys of 418 informal workers in Bogor Regency from April to May 2023 were conducted. Multivariate logistic regression analyses were performed to assess the factors associated with informal workers’ compliance with NHI contribution payments. The results revealed that being female, having lower secondary education or below, perceiving good health of family members, having negative attitudes toward and poor knowledge of the NHI, experiencing financial difficulties, preferring to visit health facilities other than public ones, and utilizing fewer outpatient services were significantly associated with the noncompliance of informal workers with NHI contribution payments. It was concluded that economic factors alone cannot contribute to informal workers’ payment compliance and that motivational factors (knowledge, attitudes toward the insurance system, and self-related health status) also encourage them to comply with contribution payments. Improving people’s knowledge, especially on the risk-sharing concept of the NHI, should be done through extensive health insurance education using methods that are appropriate for the population’s characteristics.

## 1. Introduction

Low- and middle-income countries (LMICs) have faced various obstacles in their quests to achieve universal health coverage (UHC). The ability of many health care systems to provide access to high-quality health care services has been hampered by a lack of prepayment mechanisms, as well as tools and resources to pool risks. As a result, many health systems in LMICs have relied heavily on out-of-pocket (OOP) spending to pay for medical care [1], which accounts for 30–85% of the entire cost of health care [2,3]. These countries are financially burdened by catastrophic illnesses as a result of high OOP charges [4]. It was reported that around 808 million people in 2010 had major health care spending [5], and this figure increased constantly from 2000 to 2017 [6].

Social health insurance (SHI), which involves the contribution and noncontribution schemes, is the most commonly used strategy for generating income and pooling funds to pay for medical services; this model has been proven by numerous countries that have effectively implemented UHC [7]. Positive impacts are seen after SHI implementation, including increased health care utilization [8,9,10], health quality improvements [11,12], reduction in OOP costs [13], and SHI coverage of health care expenditures [14]. However, several challenges have emerged regarding the SHI contribution scheme, such as low enrollment [15,16], adverse selection [17,18], and participant dropout [19,20], particularly among informal workers.

Indonesia has been striving to achieve UHC since 2002 and has taken further steps by implementing the family-based SHI contribution scheme called National Health Insurance (NHI; Jaminan Kesehatan Nasional (JKN)) since 2014 [19], which is the largest single-payer scheme in the world and is supervised by the Social Security Agency for Health (SSAH). In this system, the eligibility of inpatient services is divided into three classes of membership: class 1 (a contribution amount of USD 9.92 per person) for the formal sector salaried above USD 264.6 and the informal sector; class 2 (a contribution amount of USD 6.61 per person) for the formal sector salaried equal to USD 264.6 or less and the informal sector; and class 3 (a contribution amount of USD 2.77 per person) for the poor and vulnerable group or the informal sector. The contribution of the formal sector is paid proportionally (1% paid by the employee and 4% paid by the employer), that of the poor and vulnerable is paid by the government, and that of the informal sector is paid by the members themselves, except for those under class 3, for whom the government provides subsidies of about 16.6% of the contribution [21]. The details are shown in Table 1. All family members’ contributions are considered as one.

**Figure 1:**
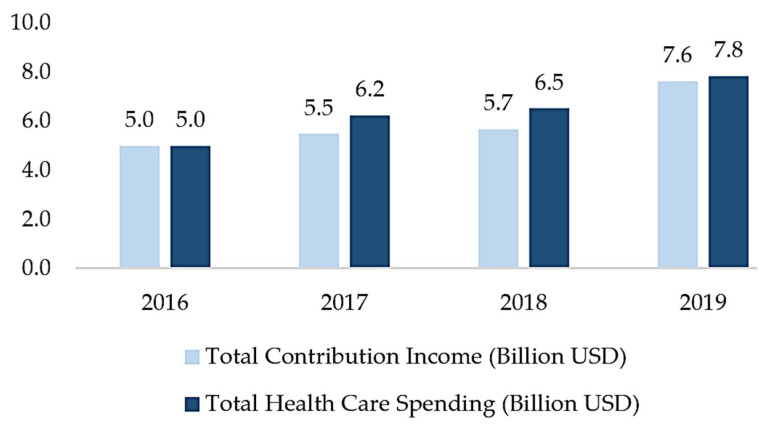
The Total Contribution Income and Health Care Spending of the NHI. Source: Adapted from Financial Aspect Social Health Security Fund 2016–2019 [26].

Following the NHI’s implementation, its coverage increased rapidly from 66.5% in 2016 to 91.7% by December 2022. The total contribution revenue increased from USD 5 billion in 2016 to USD 8.2 billion. Along with that, health care expenditure rose from USD 5 billion in 2016 to USD 7.3 billion in December 2022 [22,23]. Despite the positive effects of the program, its financial sustainability became a serious concern in 2016–2019 because of the increased number of noncompliant PBPU participants (informal workers) and the increased health care spending of the NHI [14]. In 2016–2019, there were gaps between the total revenue and the total NHI health care spending, as shown in Figure 1. In 2020–2022, this issue seemed to have abated because of the decrease in access to health care facilities. Among 12.3% of the informal workers who registered with the NHI as PBPU members (informal workers), 51% were recorded to have stopped making contributions (called noncompliant NHI contribution payment members) as of December 2022 [23]; note that the proportion of informal workers in Indonesia is 59.97% of the total working population [24]. The ratio of the average medical cost to the average contribution was reported to have increased from 115% in 2015 to 124% in 2019, which was driven by informal workers who previously did not pay their contributions regularly; this ratio is supposed to not exceed 100% [25].

Banerjee et al. [26] stated that encouraging members in the contribution scheme to pay by implementing sanctions might turn out to be a toothless mandate and only create low program enrollment, adverse selection, and a tendency to discontinue paying NHI membership, especially among informal workers [19,20]. A lack of money to pay their contributions [19,27], unaffordable contributions [20], not needing insurance again after episodes of rare illnesses (usually among the young) [28], perceived poor service quality of providers [29], forgetting to pay [20], and providers’ negative attitudes [28] are several justifications by members for their decisions to be noncompliant participants. On the other hand, Dartanto et al. [19] stated that the irregularity of informal workers’ incomes influences their economic capability more than their household monthly incomes do. The insecurity of receiving the same amount each month, even though informal employees can earn more money at once, makes it challenging for them to pay their NHI contributions, which must be made on time each month. Whereas unstable income is the main economic feature that characterizes informal workers, this has not been given adequate research attention. Therefore, in this study, the income stability of informal workers will be investigated.

For years, the SSAH has experienced financial hardships and has struggled to provide health care services because of the imbalance in revenue collection in the NHI and increased health care spending [30]. Although the government has implemented various regulations to help educate noncompliant participants and make the payment of arrears convenient for them, these have not had a significant impact, and the number of noncompliant participants has continued to rise. Informal workers who registered as NHI participants (called PBPU members) but did not comply with contribution payments will be nonactive participants. They are unable to obtain the necessary health care services unless they pay their arrears, yet they remain NHI members, as their membership is valid for life. An OOP payment could be a second option besides paying the arrears for the required health care services.

Moreover, characteristics relating to family members’ perceived health risks were also not fully investigated in previous studies. Research has measured these factors at the individual level [19,31,32], while the health insurance scheme has been studied at the family level. Having one or more family members with health risks might motivate the participants to pay their NHI contributions regularly [29,32] because of a greater demand for health insurance, which is a solution to the increasing access to formal care and increased OOP expenses [33]. By exploring the factors related to the health risks faced by family members, this study aims to investigate the determinants of compliance with NHI contribution payments among informal workers in Indonesia.

The theory of motivation–opportunity–ability (MOA) by Folke Olander and John Thogersen [34] was adapted in this study to describe the association between independent variables and the compliance of the participants with paying their NHI contributions. The MOA theory was designed based on the basic psychological concepts of motivation (encouragement toward a behavior), ability (the qualities and knowledge required for behavioral performance), and opportunity (context- and situation-specific limitations that affect how the behavior is performed) [35]. This is a suitable framework for investigating consumer behavior that affects the environment [34]. Currently, this is a commonly used method in behavioral change interventions [36]. The ability and opportunity to engage in an action then determine whether and to what extent motivation is translated into conduct [35].

## 2. Materials and Methods

### 2.1. Study Design and Participants

This research was conducted as a quantitative cross-sectional study involving face-to-face surveys from April to May 2023. The questionnaires were modified from previous studies [15,19,34].

**Figure 2:**
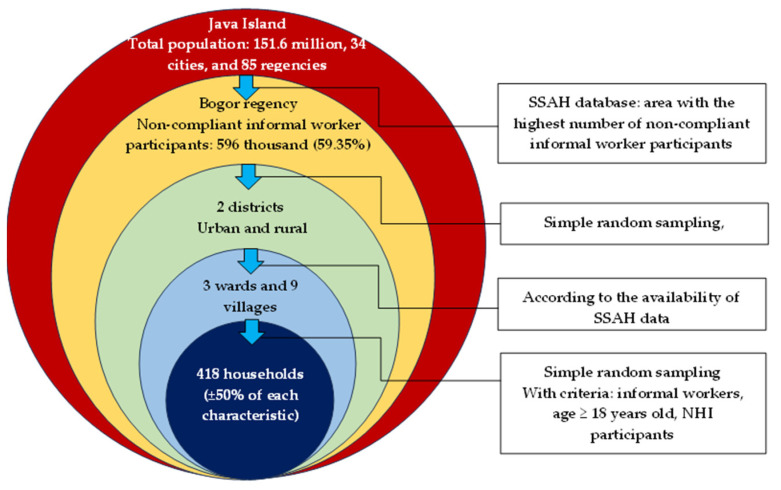
Sampling flow of the study.

Figure 2 depicts the methodology for selecting the study area, which is Java Island. In selecting the region, we obtained preliminary data on noncompliant informal workers in all areas of Java Island from the SSAH office, and we determined the area with the highest number of noncompliant informal workers. Consequently, Bogor Regency was selected among 34 cities and 85 regencies, with a total of 596,000 noncompliant informal workers among 1 million informal workers (59.35%). Then, two districts in urban and rural areas were randomly selected. Depending on the availability of the SSAH office, three wards and nine villages were included in the study as well. The list of informal workers from the SSAH database was subsequently synchronized with the local government database. A stratified sampling method of approximately 50% of each group (compliance and noncompliance) was utilized to obtain the representative sample. Finally, participants (as a household unit) were chosen randomly.

The formula proposed by Rea [37] was used to determine the sample size. A 95% confidence interval with a 5% (MEp) allowable error was selected. The level of noncompliance was set at 50% to enable the collection of a large sample size (p). The sample size was increased by 10% to account for nonresponding participants, resulting in 422 people in the final sample size. Informal workers who had registered as PBPU NHI participants, were at least 18 years old, and were willing to participate in the survey with written consent were included in this.

### 2.2. Variable Measurement

#### 2.2.1. Compliance with Payment of NHI Contributions

The participants’ compliance with paying their NHI contributions was reflected in their NHI membership statuses, which were either active or nonactive. Those participants who registered for the NHI and paid their contributions had an active NHI status and were considered compliant. On the contrary, those who registered but did not pay had a nonactive NHI membership status and were considered noncompliant. The validation of the informal workers’ NHI membership statuses was conducted using the data list of informal workers and their NHI membership statuses provided by the BPJS Kesehatan or the mobile JKN application.

#### 2.2.2. Sociodemographic Characteristics

In this section, six questions pertaining to the participants’ general information, including their living areas (urban or rural), ages, genders, education, occupations, and family sizes, were asked.

#### 2.2.3. Motivational Factors

There are three subsections under the heading of motivation factors. First, the perceived health risks faced by family members consisted of five questions adapted from Ashagrie et al. [32], including the self-rated health status, the presence of family members suffering from chronic diseases, recent illness history, the presence of older individuals in the family, and the presence of children under five years of age. The self-rated health status in this section included the health status of all family members, scaled from very poor (scored as 1) to very good (scored as 5), with the average total score used for categorizing. This was based on the consideration of the possibility that another family member’s health status might affect the decision to pay the contribution, given that the contribution payment is family-based. Second, seven scaled statements, modified from Kaso et al. [38], were used to measure the perceived quality of service received from providers, with a Cronbach’s alpha of 0.89. Lastly, attitude toward NHI comprised six Likert scale statements, adapted from a previous study [38], with a Cronbach’s alpha of 0.91. The medians of the total score were used to categorize both variables.

#### 2.2.4. Opportunity Factors

Opportunity factors consist of two subsections. First, access to health facilities was divided into two categories: less than 30 min and 30 min or above, according to the average walking velocity of young adults [39]. Second, health care service utilization was composed of five questions related to treatment-seeking behavior, the types of health facilities visited, the outpatient and inpatient services availed of in the last 12 months [40], and the cost incurred prior to joining the NHI, which were adapted from Eseta et al. [41].

#### 2.2.5. Ability Factors

Ability factors have two subsections. Knowledge of the NHI program was assessed with 10 questions related to the program covering the domains of the basic principle of the NHI’s implementation, the aim of the NHI, the contribution amount, the inpatient fine charge, the benefit package, the cost-sharing payment concept, and the risk-sharing concept, which were all adapted from previous studies [38,42]. A correct answer obtained a score of 1, and an incorrect answer obtained a score of 0. Income stability was measured using closed-ended binary questions related to the determination of income received each month and the experience of having financial difficulties.

### 2.3. Data Collection

Face validity and content validity were used to validate the questionnaire. Four experts in related fields evaluated the meaning, difficulty, and ambiguity of each questionnaire item. The item–objective congruence (IOC) score was calculated, and a score equal to or greater than 0.5 was considered acceptable. One question related to the participants’ NHI card numbers was removed because of an IOC score < 0.5. In addition, a pretest of 30 informal worker participants in another area with characteristics similar to those of the participants in the study area was conducted to measure the reliability of the questionnaire.

Afterwards, with the approval of the Committee for Research Ethics of Mahidol University Social Science Independent Review Board (COA No. 2023/059.1904), a data request support letter for informal workers registered as NHI participants in the relevant department in BPJS Kesehatan (SSAH) was issued. The list of informal worker participants was provided by the Cibinong branch office of BPJS Kesehatan and was held by NHI cadres to maintain confidentiality. Their addresses and availability were subsequently synchronized with the local government’s data. Four trained research assistants with bachelor’s degrees, accompanied by NHI cadres, collected the data through face-to-face interviews using paper-based questionnaires. Before the data collection, the objectives of the study were briefly described, and the respondents’ agreement to participate in the survey was obtained by asking them to sign written consent letters. They were assigned codes with numbers according to their order on the respondent list. All data collected were rechecked daily for completeness, and some inappropriate data were confirmed using the mobile JKN application.

### 2.4. Statistical Analysis

The Statistical Package for the Social Sciences version 21 (IBM Corp., Armonk, NY, USA) was used for the data analysis. The results were presented as numbers, percentages, means, medians, standard deviations or quartile deviations, and ranges. The associations between the determinant factors and the compliance of the respondents with paying their premiums were assessed using chi-square tests. Furthermore, the predictors of the outcome variable were evaluated through multivariate logistic regression at a 0.05 significance level. Multicollinearity among independent variables was determined by measuring the coefficient value of the Spearman correlation test. A coefficient value less than 0.75 indicated that there was no strong association between the independent variables.

## 3. Results

### 3.1. Descriptive Results of the Independent Variables

A total of 431 participants from three wards in Cibinong District (an urban area) and nine villages in Bojonggede District (a rural area) took part in the surveys. About 418 complete questionnaires were entered into the data analysis, and 13 incomplete ones were excluded. The results showed that 51.7% of the participants reported not complying with paying their NHI contributions, while their counterparts accounted for 48.3%.

Table 2 illustrates the sociodemographic characteristics of the participants. Half (50.5%) of the participants lived in rural areas, and the other half (49.5%) lived in urban areas. Most of the participants were in the 36–45 years age group (38.5%), were males (62.9%), and completed education higher than the lower secondary level (71.3%). Participants who worked in the service and industry sectors accounted for 46.7% and 14.4% of the sample, respectively, whereas 39.0% worked in other sectors. More than half of the participants (58.1%) had family sizes equal to four or more family members.

As shown in Table 3, according to the perceived health risks faced by family members, less than one-third of the participants reported having a poor health status (27.0%), while more than two-thirds reported that their families were healthy (73.0%). The majority of the respondents did not have family members who had experienced illnesses recently and who had chronic diseases (88.3% and 80.4%, respectively). Only a few (9.1%) had one or more older individuals in their families. Similarly, less than one-third (23.4%) of the participants had one or several children under five years of age in their families.

Overall, the participants indicated having good perceptions of the quality of services received from health care providers (67.9%), positive attitudes toward the NHI (53.6%), and a good understanding of the NHI program (68.2%). The details are shown in Table 4. Similarly, in the variable of perceived quality of services received from providers, nearly half of the participants (42.6%) were unsure about their satisfaction with the waiting time at outpatient services, while most of them agreed with the six other positive statements (Table 5). In terms of their attitudes toward the NHI, around one-third (31.8%) reported not being sure about improvements in drug availability after the NHI’s implementation (Table 6). None of the participants strongly disagreed with each statement about the perceived quality of services received from providers and about their attitudes toward the NHI. Regarding their knowledge about the NHI program, less than half of the respondents were able to correctly answer a question related to service coverage in contract and noncontract health facilities (34.7%), whereas for the other questions, the majority of them were able to answer correctly (Table 7).

The majority of the participants admitted to not having fixed amounts of monthly income (89.7%) and having experienced financial difficulties (77.5%). For the health care service utilization factors, more than three-quarters of the participants (75.6%) could access health facilities from their homes in less than 30 min, while 24.4% of them took 30 min or more. Most of the participants visited health facilities if they were sick (84.7%) and preferred to choose public ones (58.1%). Even so, only a few of them utilized outpatient services for more than once (15.6%) and inpatient services for one or more times (11.0%). About 89.5% of the participants admitted spending equal to IDR 1 million or less for inpatient services prior to joining the NHI (Table 8).

### 3.2. Association between the Independent Variables and Compliance with the NHI Contribution Payments

The result showed that none of the independent variables had a coefficient value greater than 0.75. The results of the chi-square tests, bivariate analysis, and multivariate analysis are shown in Table 9. Chi-square tests were conducted to assess the associations between the independent variables and the participants’ compliance with contribution payments to the NHI. Furthermore, multicollinearity among the independent variables was tested using the Spearman correlation test. The coefficient value of the Spearman correlation test should not be greater than 0.75. The results showed that none of the independent variables had coefficient values greater than 0.75.

The final multivariate logistic regression results indicated that female informal workers were 6.56 times (*p* < 0.001) less likely to comply with paying their NHI contributions compared with their counterparts. Informal workers who completed lower secondary education or below were found to be 7.52 times less likely to comply than those who completed higher education levels (*p* = 0.001). Being healthy influenced the noncompliance of informal workers with paying their NHI contributions 5.18 times (*p* = 0.007) greater than that of informal workers with a poor health status. Furthermore, having negative attitudes toward the NHI and poor knowledge of the NHI program contributed to the noncompliance of informal workers with paying their NHI contributions about 2.66 times (*p* = 0.050) and 4.94 times (*p* = 0.004), respectively. Those who had experienced financial difficulties had higher probabilities of being less likely to comply of about 4.64 times (*p* = 0.005). Likewise, informal workers who preferred to go to other types of health facilities rather than public ones to seek treatment were 4.55 times (*p* = 0.001) less likely to comply with paying their NHI contributions. Participants who never utilized or utilized outpatient services only once in the last 12 months had 8.35 times (*p* < 0.001) lower likelihood of complying with the payment of NHI contributions as opposed to those who utilized them more.

## 4. Discussion

Indonesia, an LMIC country that is heavily populated and has been working toward the achievement of UHC since 2002, implemented its NHI called JKN in 2014 as part of its ambitious NHI program; this was under the supervision of the SSAH, the largest single insurance payer in the world. Despite this, providing accessible and affordable health care services appears to be a challenge in the country because of the high percentage of informal workers who do not comply with paying their NHI contributions, thereby increasing the financial burden of the SSAH because of the imbalance between its revenue and health care spending. Therefore, determining the factors that affect informal workers’ compliance with paying their NHI contributions is important.

Based on the results of the multivariate analysis, females were 6.56 times (*p* < 0.001) less likely to comply with paying their NHI contributions compared with their counterparts; this is contrary to the finding of a study conducted in northwest Ethiopia [32], which showed that females, as a vulnerable group, were more likely to comply with paying their NHI contributions. On the other hand, Roy and Jain found that women’s financial literacy was unsatisfactory. Their lack of knowledge about insurance, savings, and investments might have influenced their perceptions and affected how they viewed the significance of maintaining active insurance [43]. Completing education at the lower secondary level or lower had a significant effect of 7.52 times (*p* < 0.001) on informal workers’ noncompliance with paying their JKN contributions compared with those who have completed education higher than the secondary level. A higher education level might indicate higher income, easier access to media to obtain information, and a more comprehensive understanding of the benefits of having health insurance [31].

As the NHI is a family-based program, we deduce that the desire to make contributions may be motivated by health issues affecting family members other than the family head. In addition, this study assessed entire families’ health statuses by utilizing their mean scores for categorization. This complements practices in the majority of similar studies, which merely measured decision makers’ health statuses; however, this might not accurately reflect a person’s motivation for the same variable. This finding implies that informal workers who assumed that their families were in good health were 5.18 times (*p* = 0.007) less likely to comply with paying their NHI contributions [44], which is consistent with the research results of Ghana [33]. As stated by Grossman [45], a person’s capacity, willingness to pay, and conviction that they need or do not need health insurance all have an impact on the demand for it. In fact, vulnerable and high-risk groups require health care more than those who are healthy [45] because of their higher risks [46], and they are more justified in purchasing more health insurance [47] to improve their access to formal care and to lower their OOP spending [33]. Conversely, it could be predicted that health insurance would be less advantageous for healthy groups, as their demand for health services would be low [44].

Furthermore, a higher probability of not complying was noted among informal workers who had negative attitudes toward the NHI, which was about 2.66 times (*p* = 0.050) greater than those who had positive attitudes toward it. Supported by previous studies [32,41] and the theory of planned behavior by Icek [48], an in-depth understanding of the NHI and trust in well-managed health insurance might foster positive attitudes toward the NHI and lead to positive actions that can make contributors to the NHI more compliant.

The existence of older individuals and children under the age of five years unexpectedly had negligible relationships with informal workers’ payment compliance. Although having senior family members may increase the health risks that families perceive, economic factors may have made it more difficult for them to make contributions [49]. Adding family members increases the cost of the required contributions, thereby exacerbating the financial burden on the entire family to make these payments. The NHI’s billing mechanism, however, requires that all contributions from a single family be made at once.

The two opportunity factors that were strongly associated with informal workers’ compliance with paying their NHI contributions were the types of health facilities used when a member of the household got sick and the frequency of outpatient service use. Informal workers were 4.55 times (*p* = 0.001) less likely to comply with paying their NHI contributions if they chose to go to health facilities (private, traditional, or drug stores) other than public ones when they were sick. According to Sharma et al. [29], people who registered at private health facilities as their first points of contact had a higher likelihood of being noncompliant. They may have decided to stop paying because of service dissatisfaction [29]. Despite the significant correlation between the types of health facilities used and informal workers’ payment compliance, this study found that the perceived quality of services received from health care providers was not statistically significant, suggesting that there may be additional aspects of the types of health facilities that affect the payment compliance of informal workers and that therefore need to be examined in future research. In addition, compared to those who used outpatient health care services more frequently, informal workers who used them less frequently in the previous 12 months had 8.35 times (*p* < 0.001) higher likelihood of failing to pay their NHI contributions. The opportunity to learn about and appreciate the value of having health insurance may have been available to those who used health care services more frequently [50]. On the other hand, a study conducted in Ethiopia [32] obtained contradictory results. Frequent visits to medical facilities could exacerbate conflicts with doctors or the staff, leading to a lack of satisfaction with the services received from providers and thus making informal workers less inclined to trust the health insurance system.

In terms of ability factors, knowledge of the NHI program and informal workers’ experiences of having financial difficulties were predictors of their payment compliance. Informal workers who had little knowledge of the program were 4.94 times (*p* = 0.004) more likely than those who knew the program well to not make the required NHI contribution payments. Similar results from previous studies support this finding [38,42]. Those who are knowledgeable about the NHI plan have a deeper awareness of its guiding principles, its benefits package, and the advantages of maintaining membership in a health insurance system [38]. Another argument could be that a thorough understanding of the NHI will improve their comprehension of the NHI notion of risk sharing [32]. Payment noncompliance among informal workers was associated with their financial difficulties roughly 4.64 times (*p* = 0.005) more than it was for those who had never experienced the same. A key finding from a previous study was that, among informal workers, income insecurity may be a contributing factor to their financial issues. The satisfaction of one’s basic needs takes precedence for those who struggle financially. Nonetheless, a study on household priorities and coping mechanisms in a context without UHC obtained a different conclusion [51]. People who had experienced deaths in the family or had sick family members said that while money can be earned, life is precious and could not be replaced, even if they had experienced financial troubles and had less income. If they have any sick family members and their NHI statuses are not active, they will do everything they can to find the money to pay their contributions or to travel to the health facility and obtain care for their sick family members. This implies that factors other than money impact people’s decisions to obtain health insurance. Even if they do not have adequate funds to pay for their NHI contributions, the terrible experience of illness or death in the family and the financial assistance provided by family and friends may be crucial elements that encourage them to maintain their health insurance at all costs.

This study also had several limitations. Regarding the diversity of population characteristics in Indonesia, this research employed a small sample size and was only carried out in one region, whose results could not be generalized and did not represent the overall population of informal workers. Thus, future research is suggested to focus on various regions with large sample sizes. Next, the lengths of months with arrears were not specified in this study, and different lengths of time of lapsed payments might indicate different reasons for not paying NHI contributions. Hence, it would be interesting to obtain more comprehensive data by including the length of months with arrears. An initial dataset related to health care service utilization is also suggested for inclusion in subsequent studies to reduce recall bias, which emerged in this study. Lastly, this research did not evaluate home expenses or monthly incomes of informal employees which might have a significant association with compliance with payment of NHI contributions. Future research which can include a variable of home expenses is recommended. Despite these limitations, this study provided novel insights by examining the perceived health risks faced by family members. We analyzed each family member’s health status as a motivation to comply with the payment of contributions, which enriches previous findings that mostly focused on assessing respondents’ health statuses only.

## 5. Conclusions

Indonesia has been struggling to maintain the NHI program’s sustainability in order to achieve UHC since informal workers do not comply with the contributions stated in the current study. The findings show that, despite the fact that irregularity of income is a significant influencing factor, motivational factors and ability-related factors were simultaneously identified, which influenced the behavior of the informal workers to be noncompliant in paying the NHI contribution. The informal workers were less inclined to make the NHI contribution when they believed that their families were in good health, which showed a weak willingness to share the risk with those who were ill. Instead, only informal workers who utilized the health care services more frequently were more likely to comply with the NHI contribution payment than those who used them less frequently, which may indicate adverse selection. Hence, a thorough understanding of the risk-sharing concept of the NHI’s participants should be taken into account to be improved by the government to raise awareness of the noteworthiness of maintaining the NHI program using appropriate methods derived from the characteristics of the population. Expanding the NHI cadre’s role from simply collecting the contribution to also educating NHI’s participants through a face-to-face approach for those who are poorly educated may be more practical.

## Figures and Tables

**Table 1:** Types of NHI Memberships.

Segment	Contribution Contributor	Contribution per Month	Benefit (Accommodation)
Poor and vulnerable groups subsidized by the central government	Central government (taxes and other funding sources)	IDR 42,000 (equal to USD 2.77)	Class 3
Poor and vulnerable groups subsidized by the local government (local government PBI)	Local government (taxes and other fund sources)	IDR 42,000 (equal to USD 2.77)	Class 3
Civil servants	Employer (government) and employees	5% of fixed income: 4% from the employer and 1% from the employee. Covers a maximum of three children. Income ceiling: IDR 12 million (equal to USD 774)	Grades 1 and 2: class 2 Grades 3 and 4: class 1
Private formal employees	Employer and employees (private company)	5% of fixed income: 4% from the employer and 1% from the employee. Covers a maximum of three children. Income ceiling: IDR 12 million (equal to USD 774)	Income ≤ IDR 4 million (equal to USD 254.6): class 2 >IDR 4 million (equal to USD 264.6): class 1
Nonemployees: retirees and veterans	Employer (government or private company) and employees	5% of the basic pension: 4% from the employer and 1% from the employee	Retirement grades 1 and 2: class 2 Retirement grades 3 and 4: class 1
Informal workers and nonworkers	Classes 1 and 2: Informal workers and nonworkers. Class 3: government and informal workers or nonworkers	Class 1: IDR 150,000 (equal to USD 9.92) Class 2: IDR 100,000 (equal to USD 6.61) Class 3: IDR 7000 or 16.6% of the total contribution Class 3: (government) IDR 35,000 (equal to 2.29 USD) (informal workers or nonworkers)	Class 1 Class 2 Class 3

Source: Adapted from the President of The Republic of Indonesia Regulation Number 82 of 2018 Concerning Health Insurance [21].

**Table 2:** Sociodemographic characteristics (n = 418).

Characteristics	Frequency (n)	Percentage (%)
Area		
Rural	211	50.5
Urban	207	49.5
Age (years)		
18–35	100	23.9
36–45	161	38.5
>45	157	37.6
Gender		
Male	263	62.9
Female	155	37.1
Education level		
Primary school or less	54	12.9
Lower secondary school	66	15.8
Higher education	298	71.3
Occupation		
Industry sector	60	14.4
Service sector	195	46.7
Others	163	39.0
Family size (members)		
<4	175	41.9
≥4	243	58.1

**Table 3:** Perceived health risk faced by family members.

Characteristics	Frequency (n)	Percentage (%)
Self-rated health status		
Poor	113	27.0
Good	305	73.0
Recent illness experience		
No	369	88.3
Yes	49	11.7
The presence of family member with a chronic disease		
No	336	80.4
Yes	82	19.6
The presence of elderly people		
None	380	90.9
One or several	38	9.1
The presence of children under the age of five		
None	320	76.6
One or several	98	23.4

**Table 4:** Participants’ perceptions of the quality of services received from providers, attitudes toward the NHI, and knowledge of the NHI program (n = 418).

Items	Number (n)	Percentage (%)
Perceived quality of services received from providers		
Poor	134	32.1
Good	284	67.9
Median 26, Q.D 1.5, Range 17–35		
Attitudes toward the NHI		
Negative	194	46.4
Positive	224	53.6
Median 24, Q.D 1, Range 13–30		
Knowledge of the NHI program		
Poor	133	31.8
Good	285	68.2
Median 8, Q.D 1.5, Range 1–10		

**Table 5:** Perceived quality of services received from providers (n = 418).

Items	Disagree n (%)	Neutral n (%)	Agree n (%)	Strongly Agreen (%)
Health service providers provide equal treatment to NHI and non-NHI participants.	39 (9.3)	61 (14.6)	312 (74.6)	6 (1.4)
The physician visits you or your family frequently when you or your family members are hospitalized.	6 (1.4)	105 (25.1)	303 (72.5)	4 (1.0)
The physician clearly describes your or your family member’s disease (the cause and the treatment process).	5 (1.2)	76 (18.2)	333 (79.7)	4 (1.0)
The physician is available according to the polyclinic schedule.	13 (3.1)	81 (19.4)	322 (77.0)	2 (0.5)
Health providers’ staff (physicians, nurses, administration officers, and other hospital staff) serve you or your family in a friendly way.	3 (0.7)	133 (31.8)	278 (66.5)	4 (1.0)
The waiting time from registration until the physician serves you at the outpatient service is satisfactory.	109 (26.1)	178 (42.6)	128 (30.6)	3 (0.7)
Providers’ staff give clear information (instructions to obtain services) and handle your or your family’s needs well.	1 (0.2)	148 (35.4)	266 (63.6)	3 (0.7)

**Table 6:** Attitudes toward the NHI (n = 418).

Items	Disagree n (%)	Neutral n (%)	Agree n (%)	Strongly Agree n (%)
The NHI makes health care affordable.	1 (0.2)	44 (10.5)	363 (86.8)	10 (2.4)
The NHI reduces the burden of health care spending.	1 (0.2)	42 (10.0)	364 (87.1)	11 (2.6)
The NHI increases access to health care services.	0 (0.0)	54 (12.9)	357 (85.4)	7 (1.7)
The quality of services received from providers has improved after the NHI’s implementation.	17 (4.1)	91 (21.8)	305 (73.0)	5 (1.2)
The availability of drugs at the health facilities has improved after the NHI’s implementation.	38 (9.1)	133 (31.8)	244 (58.4)	3 (0.7)
The NHI Committee (SSAH) manages pooled funds efficiently.	10 (2.4)	87 (20.8)	318 (76.1)	3 (0.7)

**Table 7:** Knowledge of the NHI program.

Items	Correct	
	Number (n)	Percentage (%)
The NHI is founded on the principle of mutual cooperation.	388	92.8
The aim of the NHI is to protect people from falling into poverty if they suffer from diseases, especially diseases with high costs.	392	93.8
The monthly contribution of the third class of NHI is IDR 35,000, that of the second class of NHI is IDR 100,000, and that of the first class of NHI is IDR 150,000.	364	87.1
The monthly contribution amount must be paid no later than the 10th day of each month.	348	83.3
People will be charged with inpatient service fees within 45 days after contributions or arrears are paid.	294	70.3
The NHI covers health services from contract and noncontract providers.	145	34.7
The NHI covers simple (e.g., upper respiratory tract infection, fever, and headache) and complex diseases (e.g., open heart surgery and chemotherapy), whether outpatient or inpatient services.	376	90.0
Cosmetic surgery, services that are not in accordance with the provisions, self-defeating diseases, and occupational diseases are outside the coverage of the NHI.	241	57.7
Cost sharing is done with executive clinics and in room upgrades for inpatient health services.	242	57.9
If you never use the NHI to receive treatment, your contributions will not be reimbursed.	382	91.4

**Table 8:** Income stability and health care service utilization.

Characteristics	Frequency (n)	Percentage (%)
**Income stability**		
Have a fixed amount of income per month?		
No	375	89.7
Yes	43	10.3
Have experienced financial difficulties?		
No	94	22.5
Yes	324	77.5
**Health care service utilization**		
Distance to reach health care facilities (in minutes)		
<30 min	316	75.6
≥30 min	102	24.4
Median 20, Q.D 5, Range 5–120		
Treatment-seeking behavior		
No	64	15.3
Yes	354	84.7
Types of health facilities visited		
Public	243	58.1
Private	138	33.0
Others	37	8.9
Outpatient service utilization in the last 12 months		
Never or once	353	84.4
More than once	65	15.6
Inpatient service utilization in the last 12 months		
Never	372	89.0
Once or more	46	11.0
Cost incurred for inpatient service prior to joining the NHI		
IDR ≤ 1 million	374	89.5
IDR > 1 million	44	10.5

**Table 9:** Multiple logistic regression of the association between the independent variables and informal workers’ compliance with the NHI contribution payment (n = 418).

Variables	Payment Compliance		COR a (95% CI) c	*p-*Value	AOR b (95% CI)	*p-*Value
	Compliance (%)	Noncompliance (%)				
Age (years)						
18–35	32.0	68.0	2.69 (1.59–4.54)	0.001		
36–45	55.9	44.1	1			
>45	51.0	49.0	1.22 (0.78–1.89)	0.377		
Sex						
Male	65.8	34.2	1		1	
Female	18.7	81.3	8.35 (5.18–13.46)	<0.001	6.56 (2.59–16.61)	<0.001
Education level						
Lower secondary or below	5.8	94.2	30.56 (13.73–68.00)	<0.001	7.52 (2.39–23.57)	0.001
Higher education	65.4	34.6	1		1	
Self-rated health status						
Poor	82.3	17.7	1		1	
Good	35.7	64.3	8.36 (4.88–14.30)	<0.001	5.18 (1.55–17.30)	0.007
The presence of family member with a chronic disease						
No	43.5	56.5	2.80 (1.67–4.68)	<0.001		
Yes	68.3	31.7	1			
The presence of elderly people						
None	52.1	47.9	1			
One or several	10.5	89.5	9.24 (3.21–26.56)	<0.001		
The presence of children under the age of five						
None	51.9	48.1	1			
One or several	36.7	63.3	1.85 (1.16–2.95)	0.009		
Perceived quality of services received from providers						
Poor	29.1	70.9	3.28 (2.11–5.09)	<0.001		
Good	57.4	42.6	1			
Attitude towards NHI						
Negative	32.5	67.5	3.40 (2.27–5.09)	<0.001	2.66 (1.00–7.11)	0.050
Positive	62.1	37.9	1		1	
Knowledge of NHI						
Poor	14.3	85.7	10.76 (6.25–18.52)	<0.001	4.94 (1.66–14.67)	0.004
Good	64.2	35.8	1		1	
Fix income						
No	53.1	46.9	1			
Yes	7.0	93.0	15.07 (4.58–49.58)	<0.001		
Experienced financial difficulties						
No	84.0	16.0	1		1	
Yes	38.0	62.0	8.60 (4.74–15.61)	<0.001	4.64 (1.59–13.56)	0.005
Distance of health facilities (minutes)						
< 30	55.1	44.9	1			
≥ 30	27.5	72.5	3.23 (1.98–5.27)	<0.001		
Treatment-seeking behavior						
No	20.3	79.7	4.49 (2.36–8.55)	<0.001		
Yes	53.4	46.6	1			
The type of health facilities visited						
Public	67.9	32.1	1		1	
Others	23.4	76.6	6.95 (4.48–10.77)	<0.001	4.55 (1.92–10.77)	0.001
Outpatient services utilization in the last 12 months						
Never or once	29.4	70.6	8.10 (5.15–12.73)	<0.001	8.35 (3.02–23.06)	<0.001
More than once	77.1	22.9	1		1	
Inpatient services utilization in the last 12 months						
Never	45.4	54.6	3.04 (1.55–5.97)	0.001		
Once or more	71.7	28.3	1			

Notes: a = crude odds ratio, b = adjusted odds ratio, c = confidence interval.

## Data Availability

The corresponding author may provide the data that were utilized to support the conclusions of the current study upon request.
